# Primary care transformation in Scotland: a comparison of two cross-sectional national surveys of GPs’ views in 2018 and 2023

**DOI:** 10.3399/BJGP.2024.0500

**Published:** 2025-06-02

**Authors:** Eddie Donaghy, Kieran D Sweeney, Lauren Ng, Holly Haines, Alexandra Thompson, David Henderson, Harry HX Wang, Andrew Thompson, Bruce Guthrie, Stewart W Mercer

**Affiliations:** PhD, Usher Institute, College of Medicine and Veterinary Medicine, University of Edinburgh, Edinburgh; PhD, Usher Institute, College of Medicine and Veterinary Medicine, University of Edinburgh, Edinburgh; PhD, Usher Institute, College of Medicine and Veterinary Medicine, University of Edinburgh, Edinburgh; PhD, Usher Institute, College of Medicine and Veterinary Medicine, University of Edinburgh, Edinburgh; PhD, Usher Institute, College of Medicine and Veterinary Medicine, University of Edinburgh, Edinburgh; PhD, Usher Institute, College of Medicine and Veterinary Medicine, University of Edinburgh, Edinburgh; School of Public Health, Sun Yat-Sen University, Guangzhou, China; School of Social and Political Science, University of Edinburgh, Edinburgh; PhD, Usher Institute, College of Medicine and Veterinary Medicine, University of Edinburgh, Edinburgh

**Keywords:** contracts, general practice, Scotland

## Abstract

**Background:**

The 2018 Scottish GP contract established GP clusters and multidisciplinary team (MDT) expansion. Qualitative studies have suggested suboptimal progress with these initiatives.

**Aim:**

To quantify progress since the introduction of the new contract.

**Design and setting:**

A cross-sectional postal survey of all qualified GPs was undertaken in Scotland in 2023.

**Method:**

GPs’ working lives, career intentions, and views on the new contract were compared with a similar survey conducted in 2018.

**Results:**

In total, 1385/4529 (31%) GPs responded to the 2023 survey compared with 2465/4371 (56%) in 2018. Job satisfaction and negative job attributes were similar in both surveys. Both positive job attributes (*P* = 0.011) and job pressures (*P* = 0.004) increased but the changes were small (effect sizes <0.2). Significantly more GPs were planning to reduce hours (*P*<0.001) and leave direct patient care (*P* = 0.008) in 2023 than in 2018. Quality leads’ views on cluster working were unchanged, with 70%–80% reporting insufficient support. Cluster knowledge and engagement was unchanged but there were small increases in knowledge of quality improvement. More than half of the GPs reported that access to MDT staff was insufficient to reduce their workload in all staff categories except vaccinations. Significantly more practices were trying to recruit GPs (*P*<0.01), and GPs reported worsening NHS services, higher workload, and lower practice sustainability in 2023 (*P*<0.001). Only 5% of GPs in the 2023 survey thought that the new contract had improved the care of patients with complex needs.

**Conclusion:**

GPs report few improvements in working life 5 years after the new contract was introduced, and are responding by planning to reduce their hours or leave direct patient care.

## Introduction

Internationally, ageing populations, rising multimorbidity, austerity, and widening inequalities are posing major challenges to health services.^1^^,^^2^ General practice is fundamental to addressing these challenges.^3^ Strong general practice is associated with lower health inequalities and healthcare costs^4^^,^^5^ yet it is facing unprecedented crises globally.^6^ There are concerns about the future of general practice in the UK, with shortages of GPs, declining continuity of care, and reduced access.^7^ Internationally, responses to these challenges include reforms to manage increased demand while improving efficiency,^8^ although frequently without appropriate investment.^9^ A recent systematic scoping review found expansion of multidisciplinary teams (MDTs) was the most common reform in Organisation for Economic Co-operation and Development (OECD) countries.^10^

In 2014, the Scottish Government introduced legislation to integrate health and social care services, leading to the formation in 2016 of integrated authorities (IAs), and their delivery arm, Health and Social Care Partnerships (HSCPs).^11^ In April 2018, the first-ever Scottish GP contract was introduced,^12^ although elements of it began in 2016 when the Quality and Outcomes Framework was abolished and GP clusters were introduced. Clusters are geographical groups of 5–8 practices working together to improve their local populations’ quality of care (intrinsic role) and provide local leadership within the IAs and HSCPs (extrinsic role).^12^^,^^13^ Each practice has a Practice Quality Lead (PQL), and each cluster a Cluster Quality Lead (CQL). Clusters were expected to be functional by April 2017.^14^^,^^15^ The 2018 contract aimed to reduce GP workload by expanding the MDT workforce, allowing GPs to focus on patients with complex needs as expert medical generalists.^12^^,^^13^ As of March 2023, more than 4700 whole-time equivalent new MDT staff were working in primary care in Scotland.^16^

A 2018 survey of GP clusters in Scotland conducted by the Scottish School of Primary Care reported a lack of support and training,^17^ similarly echoed in subsequent qualitative interviews in 2020–2021.^18^^,^^19^ Key barriers to effective cluster working included lack of time, poorly developed relationships, and limited data. Further interviews in 2022 with GPs and MDT staff found no perceived reduction in GP workload nor improvement in the care of patients with complex needs.^20^ MDT staff reported challenges in building new relationships, adapting to patient complexity, and the fast pace of primary care. Issues over MDT line management, training, and professional development needs were also highlighted.

**How this fits in**

Scotland introduced a new GP contract in 2018, which aimed to improve local quality of care and integration through the formation of GP clusters, and reduce GP workload by expansion of the multidisciplinary team (MDT). Qualitative studies have suggested that progress has been suboptimal but there are little quantitative data to support these suggestions. The authors quantified GPs’ working lives, career intentions, and views on the new contract progress by conducting a cross-sectional survey of all qualified GPs in Scotland in 2023 and comparing the findings with a similar survey conducted in 2018. GPs reported few improvements in working life in 2023 compared with 2018, with only small changes in cluster working. More than half reported that access to MDT staff was insufficient to reduce their workload in all staff categories except vaccinations, and significantly more were planning to reduce their hours (*P*<0.001) or leave direct patient care (*P* = 0.008) in 2023 than in 2018.

A patient survey *(n* = 1053) in 12 Scottish practices across three health boards in 2022–2023 found patients in deprived–urban areas (compared with affluent–urban or remote–rural areas) had the most complex needs, but reported the poorest experience of GP consultations.^21^ In-depth interviews with patients highlighted concerns about access, consultations length, and continuity of care.^22^ Further evaluation revealed limited patient awareness of MDT roles, and concerns about reception staff signposting to MDT care, particularly for those with complex problems in high deprivation areas.^23^

The aim of the current study was to gather GPs’ views on primary care transformation in Scotland by conducting a new national survey of views on working life, future work intentions, cluster working, and MDT expansion, and to compare these findings with the 2018 Scottish GP survey.

## Method

### Study design

Using the same process as the 2018 survey,^17^^,^^24^ a postal survey of GPs in Scotland was conducted in 2023. In both years, the survey was posted to all qualified GPs using their name and practice address.^25^ In 2023, 4529 surveys were sent in October 2023, with two reminders to non-responders. Each questionnaire contained a unique identifier, so that responders and non-responders could be identified for follow-up. Data collection stopped in early March 2024.

### Instruments used

The 2023 GP survey used the same validated measures of working life and future work intentions as the 2018 Scottish GP survey, as also used in the English National GP Worklife biennial surveys since 1999 (https://prucomm.ac.uk/), with the most recent being in 2021.^26^ The questions cover the following four domains of current working life: job satisfaction; job stressors; positive job attributes; and negative job attributes. Future work intentions relate to plans over the next 5 years to increase hours, decrease hours, continue medical work but outside the UK, leave direct patient care, or leave medical work entirely (see Supplementary Box S1 for details of the questions asked and how they were scored). Additionally, data were collected on bespoke items on cluster working, also collected in 2018,^17^ and new items about MDT expansion based on the authors’ qualitative findings and a survey conducted by Public Health Scotland,^20^^,^^27^ as explained below.

### Cluster variables

As described previously,^17^ GP Quality Leads (QLs: both CQLs and PQLs) were asked about their experiences of cluster meetings and level of support provided. All GPs were asked about their knowledge of and engagement with the cluster, and how it affected their knowledge of quality improvement (QI) (Supplementary Box S2).

### MDT variables

The 2023 survey asked GPs which MDT staff they had access to in their practice, what impact these staff had on their workload, and what the advantages and disadvantages of MDT staff were. The advantages were written as free text. GPs were also asked the following: 1) what percentage of their previous clinical work was now delegated to MDT staff; 2) what percentage they felt could be safely delegated; and 3) which staff would be most important if additional investment was available: more GPs, more MDT staff, or more administrative staff (Supplementary Box S3).

### Additional questions

Information was collected on GP demographics and employment details. GPs were asked whether their practice scheduled longer GP appointments for patients with complex needs (for example, those with multimorbidity and/or mental health problems) and if they felt the new GP contract was improving care for patients with multimorbidity who were either older people or living in deprived areas. Questions from previous English GP Worklife surveys about job changes seen in the past 12 months were also asked in both years (Supplementary Box S4).

The 2023 survey questionnaire containing the questions reported in this paper is also shown in the Supplementary Information.

### Data analysis

Standard parametric and non-parametric statistics were used, depending on the type and distribution of data for each variable. *P*-values of >0.05 were regarded as statistically significant. A Forest plot was drawn for the mean scores for the four domains, which shows the effect sizes (Cohen’s *d*) of the differences between the 2023 and 2018 surveys. The free-text answers about the advantages of MDT working were analysed by five of the authors. They each individually coded the first 200 responses, and came together to discuss and agree on a coding framework. They then shared the coding of all the responses, and classified these as positive or negative.

## Results

In total, 1385/4529 (31%) GPs responded to the 2023 survey compared with 2465/4371 (56%) in 2018.^24^ The characteristics of responding GPs were very similar in both years, and were broadly nationally representative (Supplementary Table S1). However, given the differences in response rates in the two surveys, the distribution of two key individual characteristics were further explored — gender and ethnicity — by GP age, which showed a very similar distribution across age groups in both surveys (Supplementary Figure S1). The distribution of two key practice characteristics were also explored — urbanicity and deprivation — by gender, which also showed a very similar distribution by gender in both surveys (Supplementary Figure S2).

Table 1 shows the working practices of the GPs who responded to the surveys. GPs reported working significantly fewer sessions per week (*P*<0.001) and taking significantly fewer holidays per year in 2023 than in 2018 (*P*<0.001).

**Table 1:** GP working practices in 2023 and 2018

	GP survey 2023 (*n* = 1385)	GP survey 2018 (*n* = 2465)	*P*-value^a^
Mean years in current practice (SD)	12.2 (8.9)	12.5 (9.4)	0.951

Mean sessions per week (SD)	6.6 (1.6)	6.9 (1.8)	<0.001

Mean weeks holiday/year (SD)	6.5 (1.6)	6.8 (2.9)	<0.001

Current employment model (*n/*/%)			
Partnership	1143/1383 (82.6%)	2048/2445 (83.8%)	
Salaried	232/1383 (16.8%)	387/2445 (15.8%)	0.364
Locum/other	8/1383 (0.6%)	10/2445 (0.4%)	

Future employment model (*n*/%)			
Partnership	957/1215 (78.8%)	1778/2235 (79.6%)	
Salaried	203/1215 (16.7%)	351/2335 (15.7%)	0.630
Locum/other	55/1215 (4.5%)	106/2235 (4.7%)	

Actively seeking new model (*n*/%)	87/1356 (6.3%)	160/2443 (6.5%)	0.873

Does out of hours (*n*/%)	222/1375 (16.1%)	585/2457 (23.8%)	<0.001

a Non-parametric (Mann–Whitney) test was used to calculate *P*-values.

SD = standard deviation.

### Current working life and future intentions

**Figure 1:**
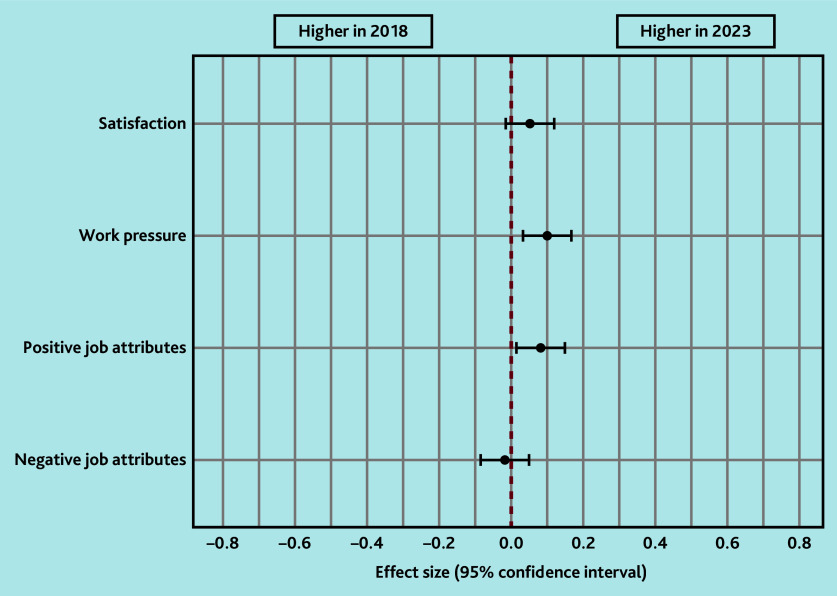
Effect size differences in working life domains in 2018 and 2023. Values that lie to the right of the red dotted vertical line mean that the scores were higher (better) in 2023 than in 2018, and those lying to the left mean the opposite. If the confidence intervals (black bars) cross the red line, then the difference was not statistically significant. It should be noted that, although statistically significant differences were found, the sizes of the differences were small (effect size <0.2).

Table 2 shows the results for the domains of job satisfaction, job pressure, job attributes, and future work intentions. Although work pressures were significantly higher (*P* = 0.004), mean positive job attributes significantly improved in 2023 (*P* = 0.011) and negative job attributes remained the same. Mean job satisfaction was similar in both survey years. However, it should be emphasised that, irrespective of *P*-values, the differences in mean scores in the 2 survey years were very small, with effect sizes of <0.2 (Figure 1).

**Table 2:** GPs’ current working life and future work intentions in 2023 and 2018^a^

	GP Survey 2023 (*n* = 1378)	GP Survey 2018 (*n* = 2465)	*P*-value
	Mean (SD)	Mean (SD)	

**Current working life:**			
Job satisfaction	5.31 (0.95)	5.26 (0.99)	0.128
Job pressures	3.52 (0.78)	3.45 (0.74)	0.004
Positive job attributes	3.34 (0.54)	3.29 (0.59)	0.011
Negative job attributes	3.85 (0.56)	3.86 (0.58)	0.594

**Future intentions:**			
*All GPs*			
Increase hours	1.41 (0.83)	1.49 (0.95)	0.090
Decrease hours	3.08 (1.56)	2.81 (1.56)	<0.001
Work outside UK	1.44 (0.92)	1.42 (0.85)	0.923
Leave direct patient care	2.39 (1.53)	2.24 (1.52)	0.008
Leave medical work entirely	2.24 (1.52)	2.18 (1.53)	0.060

*GPs aged <55 years*			
Increase hours	1.49 (0.88)	1.59 (1.03)	0.170
Decrease hours	2.74 (1.47)	2.51 (1.44)	<0.001
Work outside UK	1.48 (0.94)	1.44 (0.87)	0.874
Leave direct patient care	1.91 (1.19)	1.75 (1.11)	<0.001
Leave medical work entirely	1.72 (1.09)	1.62 (1.04)	0.002

a Satisfaction items measured on a 7-point scale (1 = extremely dissatisfied, 7 = extremely satisfied). Work pressure is measured on a 5-point scale (1 = no pressure, 5 = high pressure). Job attributes are measured on a 5-point scale (1 = strongly disagree, 5 = strongly agree). Job control, job design, work pressure, and workload are derived from job attributes (see Method). Future intentions measured on a 5-point scale: 1 = none; 2 = slight; 3 = moderate; 4 = considerable. 5 = high. *T*-tests were used to calculate the *P*-values for the working life domains as these were normally distributed. Non-parametric (Mann–Whitney) U test was used to calculate *P*-values for the future job intentions.

For individual item scores for the four domains — job satisfaction, job pressure, positive job attributes, and negative job attributes — see Supplementary Tables S2–S5.

**Figure 2:**
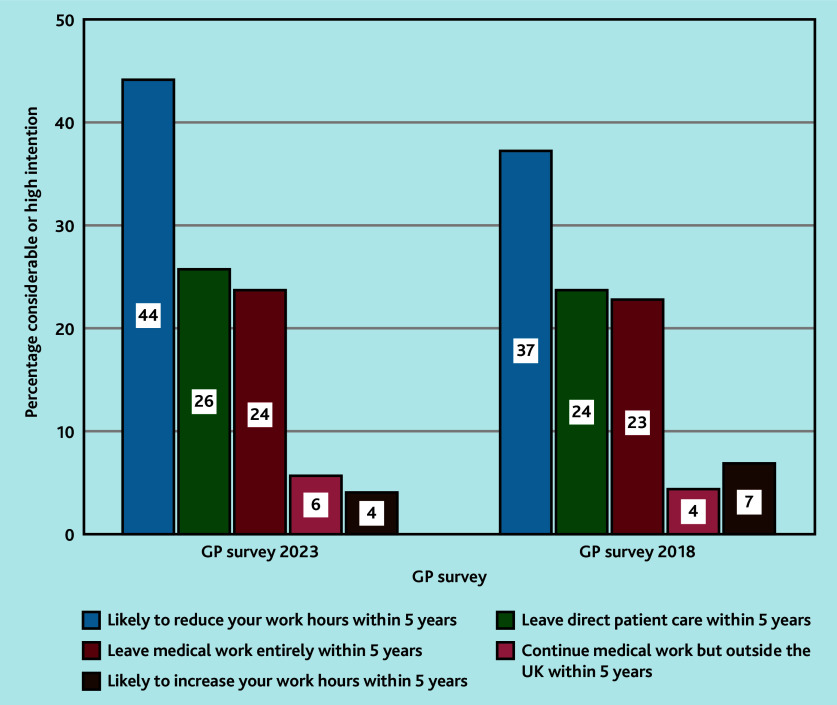
Future work intentions of GPs in 2023 and 2018. Non-parametric (Mann–Whitney) *U* test was used to calculate *P*-values for the future job intentions. The difference between likely to reduce hours in 2023 and 2018 was significant (*P*<0.001) as was the intention to leave direct patient care (*P* = 0.008).

In 2023, significantly fewer GPs intended to increase their hours, and significantly more intended to decrease hours (44% in 2023 versus 37% in 2018; *P*<0.001) or leave direct patient care entirely (26% in 2023 versus 24% in 2018; *P* = 0.008) than in 2018 (Table 2, Figure 2). For GPs aged <55 years, significantly more planned to reduce their hours (33% versus 26%; *P*<0.001), leave direct patient care (13% versus 10%; *P*<0.001), and leave medical work entirely (9% versus 8%; *P* = 0.002) in 2023 than in 2018 (Table 2).

### Clusters

QLs’ views on cluster meetings did not change between the two surveys (see Supplementary Table S6). There was no difference in the extent to which they felt supported overall with 70–80% feeling insufficiently supported, but individual items showed significantly less support for analysis (*P* = 0.006), and significantly more support for QI methods (*P* = 0.041 and leadership (*P* = 0.009) in 2023 than in 2018 (Supplementary Table S6). For all other GPs (neither CQLs nor PQLs), mean overall scores for knowledge and engagement with clusters did not differ between 2023 and 2018, but statistically significant improvements in individual items were found for ‘decisions’ (*P* = 0.003) and ‘queries’ (*P*<0.001) (Supplementary Table S6). There was a statistically significant improvement in mean overall QI score in 2023 (*P*<0.001), which reflected small increases in all six aspects of QI (Supplementary Table S6).

### Multidisciplinary teams

In 2023, GPs reported that 8.5% of workload had been delegated to MDT staff, but estimated that 22% could, in principle, be safely delegated (*P*<0.001). In 2023, 82% felt that more GPs was the most important issue for any future investment, compared with 69% in 2018 (*P*<0.001) (data not shown).

Table 3 shows the 2023 GPs’ views on new MDT staff (these questions were not asked in 2018). Access to different MDT staff varied widely. More than half of the GPs reported that access was insufficient to reduce their workload in all staff categories except vaccinations. Free-text answers about advantages of MDT expansion were provided by 84%, and 54.9% documented one positive comment, 28.9% two, and 9.8% three or more. Just under 1 in 4 (23.4%) documented both positive and negative comments.

**Table 3:** GPs’ views on availability and impact on workload of new MDT staff 2023^a^

	No access *n* (%)	Some access but insufficient to reduce my workload *n* (%)	Sufficient access to reduce my workload *n* (%)	Not sure *n* (%)
Pharmacy	16 (1.2%)	788 (56.9%)	579 (41.8%)	1 (0.1%)
Urgent care	570 (41.4%)	406 (29.5%)	390 (28.3%)	10 (0.7%)
Advanced physiotherapy	443 (32.1%)	493 (35.7%)	441 (31.9%)	5 (0.4%)
Mental health	477 (34.5%)	637 (46.1%)	265 (19.2%)	4 (0.3%)
Link workers	407 (29.5%)	575 (41.6%)	340 (24.6%)	59 (4.3%)
CTAC nurses	155 (11.2%)	606 (43.9%)	594 (43.0%)	26 (1.9%)
Vaccinations	153 (11.2%)	223 (16.3%)	834 (61.0%)	157 (11.5%)

a Percentages are valid percentages, excluding missing values.

CTAC = Community Treatment and Care Team. MDT = multidisciplinary team.

For the positive comments, themes related to the additional clinical expertise and skill mix, better collaborative teamworking, learning from MDT staff, gaining new perspectives and ideas, better links with secondary care, better links with community resources, and improved patient care and patient safety. Comments on reduced workload were often qualified as *‘some reduction’* owing to increased patient demand and insufficient MDT workforce.

The negative comments largely related to the issues in the subsequent question about the disadvantages of the new MDT, including lack of clinical space (68% agreed), the need to provide training and supervision (61% agreed), and lack of control over what the MDT staff actually do (71% agreed) (Supplementary Table S7).

### Recruitment, local NHS services, sustainability, and training

More GPs in 2023 reported that their practices were trying to recruit GPs than in 2018 (35.8% versus 30.5%; *P*<0.01), with more trying for longer than 12 months (42% versus 30.9%; *P*<0.001) (data not shown). In 2023, more GPs felt that local NHS services had significantly worsened in the past 12 months, that practice and personal workload was higher, and that the long-term sustainability of their practice was worse, compared with 2018 (all *P*<0.001) (Supplementary Table S8).

Overall, more practices were involved in the training and education of undergraduate students in 2023 than 2018 (74% versus 59%; *P*<0.001). In 2023, 72% were involved in training (non-medical) healthcare professionals, compared with 55% in 2018 (*P*<0.001) (Supplementary Figure S3).

### Improving the care of patients with complex needs

Fewer GPs reported giving longer consultations for complex patients in 2023 than in 2018 (39.8% versus 52.2%, respectively; *P*<0.001). Only 5% of GPs thought the new contract was improving the care of older patients with multimorbidity, and only 4% felt it was improving the care of patients with multimorbidity in deprived areas (data not shown).

## Discussion

### Summary

Scottish GPs’ working lives, career intentions, and views on cluster working in 2023 showed little change since 2018. Job satisfaction and negative job attributes did not change, and, although both positive job attributes (*P* = 0.011) and job pressure (*P* = 0.004) increased, the differences were very small (effect sizes <0.2) and thus unlikely to be meaningful. However, worryingly, more GPs were planning to reduce their hours and leave direct patient care in 2023 than in 2018.

Views on clusters were largely unchanged but there were small improvements in understanding of QI. More than half of the GPs reported that access to MDT staff was insufficient to reduce their workload in all staff categories except vaccinations. In addition, fewer GPs reported offering longer consultations to complex patients in 2023 than in 2018. Significantly, more practices in 2023 were trying to recruit GPs (*P*<0.01), and GPs reported worsening NHS services, higher workload, and lower practice sustainability (*P*<0.001). Only 1 in 20 GPs believed the new contract has improved care for patients with complex needs.

### Strengths and limitations

A key strength of this study is having comparable and broadly representative national data examining GP views across the first 5 years of the new Scottish GP contract. The 2023 response rate was lower than in 2018 (30% versus 56%), but the characteristics of responding GPs was virtually identical in both years, even in sub-group analysis by age and gender. Analysis of GPs who responded to both surveys generally supported our conclusions (Supplementary Tables S9 and S10).

The limited impact of the new GP contract should be considered in the context of the disruption caused by the COVID-19 pandemic.

### Comparison with existing literature

The present study’s finding that job pressure has increased is mirrored by the 2021 English GP Worklife survey,^26^ with other data from England showing a higher number of appointments being provided in 2023 than any year since 2018, despite a fall in the number of fully trained full-time equivalent GPs.^28^^,^^29^ In contrast to the present study’s finding that overall job satisfaction in Scotland did not changed from 2018 to 2023, scores on the same measure in the English GP Worklife survey fell significantly between 2019 and 2021.^26^ English data show a continued trend of GPs reducing their working hours, and increasing numbers planning to leave direct patient care,^26^^,^^29^ in keeping with the present study’s findings. The authors plan to compare the Scottish 2023 survey with the next English GP Worklife survey (2023–2024) when data become available.

The findings on MDT staff echo those of a recent Public Health Scotland survey of GPs, which reported insufficient access to MDT staff (particularly urgent care staff), and a lack of reduction in GP workload with no release of time for complex patients, and similar concerns about disadvantages.^30^ Similar findings have been reported in England in the Additional Roles Reimbursement Scheme.^31^

### Implications for research and practice

There is widespread recognition that general practice faces an unprecedented crisis, across the UK and in other high-income countries. This survey of Scottish GPs provides further evidence that the key aims of the 2018 GP contract have not been realised and, despite the rapid increase in the MDT workforce, the majority of GPs reported no decrease in workload. This has implications for ongoing negotiations for phase two of the GP contract in Scotland, highlighting the need to take more robust measures to reduce GP workload and improve workforce sustainability. It may be that the key to reducing GP workload does not lie in the expansion of the MDT but in the expansion of the GP workforce itself. In 2017 the Scottish Government pledged an additional 800 GPs within a decade,^32^ but since then GP whole-time equivalent numbers have fallen, not risen.^33^ New approaches are required to increase GP recruitment and retention. Further longitudinal research is required on routine consultation data to confirm the findings of the survey.

In conclusion, although there have been minor improvements in some aspects of GP working life in Scotland since the new contract in 2018, most aspects have remained the same or worsened. GPs — including younger GPs — are responding by planning to reduce their hours or leave direct patient care, which is a worrying picture given the problems of GP recruitment and retention.

## Supplementary Information



## References

[1] Organisation for Economic Co-operation and Development (2019). Health for everyone? Social inequalities in health and health systems.

[2] Commonwealth Fund (2023). Overworked and undervalued: unmasking primary care physicians’ dissatisfaction in 10 high-income countries: findings from the 2022 International Health Policy Survey.

[3] Royal College of General Practitioners (2012). Medical generalism: why expertise in whole person medicine matters.

[4] Macinko J, Starfield B, Shi L (2007). Quantifying the health benefits of primary care physician supply in the United States. Int J Health Serv.

[5] Starfield B, Shi L, Macinko J (2005). Contribution of primary care to health systems and health. Milbank Q.

[6] World Health Organization (WHO), Regional Office for Europe (2022). Health and care workforce in Europe: time to act.

[7] House of Commons, Health and Social Care Committee (2022). The future of general practice. Fourth report of session 2022–23.

[8] WHO, United Nations Children’s Fund (2018). A vision for primary health care in the 21st century: towards universal health coverage and the Sustainable Development Goals.

[9] Hanson K, Brikci N, Erlangga D (2022). The Lancet Global Health Commission on financing primary health care: putting people at the centre. Lancet Glob Health.

[10] Henderson DAG, Donaghy E, Dozier M (2023). Understanding primary care transformation and implications for ageing populations and health inequalities: a systematic scoping review of new models of primary health care in OECD countries and China. BMC Med.

[11] Scottish Parliament (2014). Public Bodies (Joint Working) (Scotland) Act 2014.

[12] Scottish Government (2017). The 2018 General Medical Services Contract in Scotland.

[13] Audit Scotland (2019). General Medical Services contract in Scotland: a short guide.

[14] Scottish Government (2017). Improving together: A National Framework for Quality and GP Clusters in Scotland.

[15] Scottish Government (2016). Directorate for Population Health Improvement and the BMA Scottish General Practitioners Committee: communication on supporting materials to health board chief executives, Health and Social care Partnership chief officers and practices in relation to the Transitional Quality Arrangements (TQA) for the 2016/17. General Medical Services (GMS) contract.

[16] Scottish Government (2023). Primary care improvement plans: summary of implementation progress at March 2023.

[17] Mercer S, Gillies J, Fitzpatrick B (2020). Progress of GP clusters 2 years after their introduction in Scotland: findings from the Scottish School of Primary Care national GP survey. BJGP Open.

[18] Huang H, Jefferson ER, Gotink M (2021). Collaborative improvement in Scottish GP clusters after the Quality and Outcomes Framework: a qualitative study. Br J Gen Pract.

[19] Donaghy E, Huang H, Henderson D (2023). Primary care transformation in Scotland: qualitative evaluation of the views of national senior stakeholders and cluster quality leads. Br J Gen Pract.

[20] Donaghy E, Huang H, Henderson D (2024). Primary care transformation in Scotland: a qualitative study of GPs’ and multidisciplinary team members’ views. Br J Gen Pract.

[21] Sweeney KD, Donaghy E, Henderson D (2024). Patients’ experiences of GP consultations following the introduction of the new GP contract in Scotland: a cross-sectional survey. Br J Gen Pract.

[22] Donaghy E, Sweeney K, Henderson D (2024). Primary care transformation in Scotland: a qualitative evaluation of the views of patients. Br J Gen Pract.

[23] Sweeney KD, Donaghy E, Henderson D (2024). Patients’ views on primary care multidisciplinary teams in Scotland: a mixed-methods evaluation. BJGP Open.

[24] Hayes H, Gibson J, Fitzpatrick B (2020). Working lives of GPs in Scotland and England: cross-sectional analysis of national surveys. BMJ Open.

[25] Public Health Scotland (2019). General practitioner contact details.

[26] Odebiyi B, Walker B, Gibson J (2022). Eleventh National GP Worklife Survey 2021.

[27] Public Health Scotland (2023). Primary care reforms: GP feedback survey Main report.

[28] England NHS (2024). Appointments in general practice Official statistics in development.

[29] NHS England (2024). General practice workforce. Official statistics.

[30] Public Health Scotland (2023). Primary care reform: GP feedback survey.

[31] MacConnachie V (2024). Assessing the impact and success of the Additional Roles Reimbursement Scheme. NHS Confederation, Primary Care Network.

[32] Scottish Government (2017). 800 more GPs for Scotland.

[33] Public Health Scotland (2022). General practice workforce survey.

