# Supplementary material for: Primary care transformation in Scotland: a comparison of two cross-sectional national surveys of GPs’ views in 2018 and 2023
Source: Br J Gen Pract. 2025 Jun 2:BJGP.2024.0500. Online ahead of print. doi: 10.3399/BJGP.2024.0500 (PMC12159868; doi:10.3399/BJGP.2024.0500)
Supplement: Supplementary file 1 [file BJGP.2024.0500_suppl.pdf]

Supplementary File

Box S1 – Details of working life questions

The working life survey has four related domains of working lives; job satisfaction, job stressors, positive job attributes, and negative job attributes, with each domain having a series of individual questions.[4] For each domain, we created an average score for all of the questions for each respondent.

Job satisfaction was measured by 10 different items with ratings on a 7-point scale from 1 "extremely dissatisfied" and to 7 "extremely satisfied". The 10 answers were then averaged (minimum possible score of 1 and maximum possible score of 7).

Job stressors were measured as the pressure experienced from 15 job items, rated on a 5-point scale; 1 "no pressure", 2 "slight pressure", 3 "moderate pressure", 4 "considerable pressure", 5 "high pressure". The 13 answers were averaged (minimum possible score of 1 and maximum possible score of 5).

Job attributes had eleven statements regarding 'positive' job attributes, and six statements regarding negative job attributes, where 1 indicates "strongly disagree", 2 "disagree", 3 "neutral", 4 "agree" and 5 "strongly agree". The answers for the positive and negative job attributes were separately averaged (minimum possible score of 1 and maximum possible score of 5).

GPs intentions (in the next 5 years) to increase work hours, reduce work hours, leave medical work entirely, leave direct patient care, or to continue medical work but outside the UK, were measured using a 5-point scale with 1 indicating "none", 2 "slight", 3 "moderate", 4 "considerable" and 5 "high".

Box S2 – Details of Cluster questions

As described previously [2], GP Quality Leads (QLs) – both Cluster Quality Leads (CQLs) and Practice Quality Leads (PQLs) were asked to what extent Cluster meetings were: 1) well organised; 2) friendly; 3) well facilitated; and 4) productive. They answered using a five-point response scale ('always', 'nearly always', 'sometimes', 'hardly ever', or 'never'). QLs were also asked how supported they felt in relation to: 1) data; 2) health intelligence; 3) analysis; 4) quality improvement methods; 5) advice; 6) leadership; and 7) evaluation and research. They responded on a four-point scale ('fully supported', 'almost fully supported', 'somewhat supported', or 'not at all supported'). They were also asked whether the focus was mainly on the 'intrinsic' role (quality improvement) or the 'extrinsic role' (participation in local planning of integrated care) of the Clusters.

All GPs were asked about their knowledge and engagement with the Cluster: 1) 'I feel informed about what my Cluster is trying to achieve'; 2) 'decisions made by my Cluster reflect my views'; 3) 'when I make contact, my PQL is responsive to my queries and concerns'; 4) 'my GP Cluster is "owned" by its members and feels like "our" organisation'; and 5) 'I can influence the work of my cluster if I chose to'. The GPs rated their answers on a five-point scale from 'strongly disagree;' to 'strongly agree' (mid-point being 'neutral'). All GPs were also asked how GP Clusters had affected their understanding of : 1) quality planning (how to set quality improvement goals); 2) quality improvement (methods and approaches); 3) quality control (measuring improvement, ensuring safety); 4) the characteristics of your local population of patients (such as age, deprivation, multimorbidity levels); 5) the quality of care they provide; and 6) the extent to which they involve patients in decisions about their care, based on what's important to them. The answers were measured on a five-point scale from 'decreased a lot' to 'increased a lot' (mid-point 'not changed').

Box S3 – Details of MDT questions

GPs in the 2023 survey were asked about which MDT staff they had access to in their practice, and if this was sufficient to decrease their workload, which was rated as 'no access', 'some access but insufficient to decrease my workload'; 'sufficient access to reduce my workload' and 'don't know'. They were then asked to describe the main advantages of the new MDT staff as free text. They were then asked if there had been any disadvantages of the new MDT staff in terms of clinical space, training and supervision, losing tasks they used to enjoy, and not having control over what the MDT actually do. Responses were on a seven-point Likert scale where 1 = 'strongly agree' and 7 = 'strongly disagree'.

GPs were also asked a) what percentage of their previous clinical work was now delegated to MDT staff; b) what percent they felt could be safely delegated, and; c) which staff would be most important if additional investment was available – more GPs, more MDT staff, or more administrative staff.

Box S4 – Additional Questions

GPs were asked whether their practice scheduled longer GP appointments for patients with complex needs (such as those with multimorbidity and/or mental health problems) and if they felt the new GP contract was improving the care of patients with multimorbidity who were either; a) elderly or; b) living in deprived areas. These questions were all answered as 'yes'; 'no'; or 'don't know'.

Information was also collected on GP demographics (age, gender, ethnicity), their model of employment, how long they had worked in the practice, the number of sessions they worked per week, holidays taken per year, and if they currently did out of hours work.

Questions from previous working life surveys in England were also asked (in both surveys) about changes they had seen in their job in the last 12 months in terms of NHS services locally, in their Practice, the practice workload, their personal workload, the sustainability of the practice, and their job satisfaction, on a 5 point Likert scale where 1 = 'worsened a lot' to 5 = 'improved a lot'. They were also asked if their practice was trying to recruit more GPs ('yes'; 'no'; 'don't know') and for how long (< 3 months; 3-6 months; 7-12 months; > 12 months). They were also asked their practice had been involved in training and education of a) undergraduate students; b) Foundation doctors; c) GPs in training; d) other healthcare professionals, and e) others.

Additional bespoke questions were included in the 2023 survey about social prescribing, green prescribing, and management of frail patients, which will be reported in a future paper.

2023 Survey:

THE UNIVERSITY of EDINBURGH | usher institute

2023 SCOTTISH GP WORKLIFE SURVEY

Confidentiality Statement

The number on the right will be used to manage the questionnaire responses. All information that identifies an individual or GP Practice will be held strictly confidential, will be used only for the purposes of this survey, and will not be released to other persons or used for any purpose other than this survey.

survey tracking number

If you wish to participate, please complete the questionnaire and return it to us in the enclosed pre-paid, pre-addressed envelope.

If you do NOT wish to participate and do not want to receive reminders, please return the blank questionnaire in the envelope provided.

A. About Your Employment as a GP

1. In what year did you qualify as a GP? .... (record year)

2. How long have you worked in your current, main Practice of employment? .... (record years)

3. How many sessions do you work in a typical week? .... record number

4. Please ✓ the relevant box to indicate your current, main Practice of employment and preferred model of employment in next 12 months

	Current, Main Model of Employment	Preferred Model of Employment in next 12 months
Partner/Principal	☐ 1	☐ 1
Salaried (including assistant, retainer)	☐ 2	☐ 2
Locum	☐ 3	☐ 3
Other (please specify)	☐ 4	☐ 4

5. Are you actively seeking a new employment model? Yes ☐ 1 No ☐ 2

If Yes, please specify the type of model you are seeking ....

6. Do you personally do OUT-OF-HOURS work? Yes ☐ 1 No ☐ 2

… If Yes, on average how many hours per week do you spend doing out-of-hours work? .... hours

7. How many weeks holiday do you take per year? .... weeks

B. About Your Job as a GP

1. Please ✓ the relevant box on this scale to indicate how satisfied you are with these aspects of your job.

		1 = Extremely dissatisfied						7 = Extremely satisfied
a	Physical working conditions	☐ 1	☐ 2	☐ 3	☐ 4	☐ 5	☐ 6	☐ 7
b	Freedom to choose your own method of working	☐ 1	☐ 2	☐ 3	☐ 4	☐ 5	☐ 6	☐ 7
c	Your colleagues and fellow workers	☐ 1	☐ 2	☐ 3	☐ 4	☐ 5	☐ 6	☐ 7
d	Recognition you get for good work	☐ 1	☐ 2	☐ 3	☐ 4	☐ 5	☐ 6	☐ 7
e	Amount of responsibility you are given	☐ 1	☐ 2	☐ 3	☐ 4	☐ 5	☐ 6	☐ 7
f	Your remuneration	☐ 1	☐ 2	☐ 3	☐ 4	☐ 5	☐ 6	☐ 7
g	Opportunities to use your abilities	☐ 1	☐ 2	☐ 3	☐ 4	☐ 5	☐ 6	☐ 7
h	Your hours of work	☐ 1	☐ 2	☐ 3	☐ 4	☐ 5	☐ 6	☐ 7
i	Amount of variety in your job	☐ 1	☐ 2	☐ 3	☐ 4	☐ 5	☐ 6	☐ 7
j	Taking everything into consideration, how do you feel about your job	☐ 1	☐ 2	☐ 3	☐ 4	☐ 5	☐ 6	☐ 7

2. Please ✓ the relevant box on the scale below to rate how much pressure you experience from each of these factors in your job.

		No pressure	Slight pressure	Moderate pressure	Considerable pressure	High pressure
a	Increased demands from patients	☐ 1	☐ 2	☐ 3	☐ 4	☐ 5
b	Dealing with problem patients	☐ 1	☐ 2	☐ 3	☐ 4	☐ 5
c	Dealing with earlier discharges from hospital	☐ 1	☐ 2	☐ 3	☐ 4	☐ 5
d	Worrying about patient complaints/litigations	☐ 1	☐ 2	☐ 3	☐ 4	☐ 5
e	Having insufficient time to do justice to the job	☐ 1	☐ 2	☐ 3	☐ 4	☐ 5
f	Interruptions by emergency calls during surgery	☐ 1	☐ 2	☐ 3	☐ 4	☐ 5
g	Unrealistically high expectation of role by others	☐ 1	☐ 2	☐ 3	☐ 4	☐ 5
h	Insufficient resources within the Practice	☐ 1	☐ 2	☐ 3	☐ 4	☐ 5
i	Long working hours	☐ 1	☐ 2	☐ 3	☐ 4	☐ 5
j	Paperwork	☐ 1	☐ 2	☐ 3	☐ 4	☐ 5
k	Changes to meet requirements from external bodies (such as GP Cluster, HSCP, Health Board)	☐ 1	☐ 2	☐ 3	☐ 4	☐ 5
l	Meeting requirements for quality-linked payments	☐ 1	☐ 2	☐ 3	☐ 4	☐ 5
m	Finding a locum	☐ 1	☐ 2	☐ 3	☐ 4	☐ 5
n	Adverse publicity by the media	☐ 1	☐ 2	☐ 3	☐ 4	☐ 5
o	Increasing workloads	☐ 1	☐ 2	☐ 3	☐ 4	☐ 5

3. Please ✓ the relevant box to rate the extent to which you agree/disagree with these statements.

		Strongly disagree	Disagree	Neutral	Agree	Strongly agree
a	I have a choice in deciding how I do my job	☐ 1	☐ 2	☐ 3	☐ 4	☐ 5
b	I have a choice in deciding what I do at work	☐ 1	☐ 2	☐ 3	☐ 4	☐ 5
c	My working time can be flexible	☐ 1	☐ 2	☐ 3	☐ 4	☐ 5
d	My job provides me with a variety of interesting things	☐ 1	☐ 2	☐ 3	☐ 4	☐ 5
e	I have to work very fast	☐ 1	☐ 2	☐ 3	☐ 4	☐ 5
f	I have to work very intensively	☐ 1	☐ 2	☐ 3	☐ 4	☐ 5
g	The patients I see are presenting with increasingly complex care needs	☐ 1	☐ 2	☐ 3	☐ 4	☐ 5
h	I am involved in deciding on the changes introduced that affect my work	☐ 1	☐ 2	☐ 3	☐ 4	☐ 5
i	I always know what my work responsibilities are	☐ 1	☐ 2	☐ 3	☐ 4	☐ 5
j	I am consulted about changes that affect my work	☐ 1	☐ 2	☐ 3	☐ 4	☐ 5
k	I do not have time to carry out all my work	☐ 1	☐ 2	☐ 3	☐ 4	☐ 5
l	I get clear feedback about how well I am doing my job	☐ 1	☐ 2	☐ 3	☐ 4	☐ 5
m	Relationships at work are strained	☐ 1	☐ 2	☐ 3	☐ 4	☐ 5
n	I can decide on my own how to go about doing my work	☐ 1	☐ 2	☐ 3	☐ 4	☐ 5
o	Changes to my job in the last year have led to better patient care	☐ 1	☐ 2	☐ 3	☐ 4	☐ 5
p	I am required to do unimportant tasks which prevent me completing more important ones	☐ 1	☐ 2	☐ 3	☐ 4	☐ 5
q	My patients trust my generalist professional skills	☐ 1	☐ 2	☐ 3	☐ 4	☐ 5

4. Please ✓ the relevant box on the scale below to indicate your opinions about changes you have seen in GP work in the last 12 months …

		Worsened a lot	Worsened a little	Not changed	Improved a little	Improved a lot	Don't know
a	NHS services for patients in your Practice	☐ 1	☐ 2	☐ 3	☐ 4	☐ 5	☐ 6
b	NHS services for patients in the local area	☐ 1	☐ 2	☐ 3	☐ 4	☐ 5	☐ 6
c	Practice workload	☐ 1	☐ 2	☐ 3	☐ 4	☐ 5	☐ 6
d	Your personal workload	☐ 1	☐ 2	☐ 3	☐ 4	☐ 5	☐ 6
e	Long term sustainability of your Practice	☐ 1	☐ 2	☐ 3	☐ 4	☐ 5	☐ 6
f	Your job satisfaction	☐ 1	☐ 2	☐ 3	☐ 4	☐ 5	☐ 6

C. About the new Scottish GP contract

A) Expansion of the Multidisciplinary Team (MDT)

There is ongoing expansion of the multidisciplinary team (MDT) in general practice such as clinical pharmacists, advanced nurse practitioners, MSK physiotherapists, paramedics, link workers and others. Please answer the questions on this topic below:

1. Which of the following MDT staff does your (main) Practice currently have access to and is this sufficient to reduce your workload?

		No access	Some access, but insufficient to reduce my workload	Sufficient access to reduce my workload	I am not sure
a	Pharmacy staff	☐ 1	☐ 2	☐ 3	☐ 4
b	Urgent care staff (e.g. advanced nurse practitioners)	☐ 1	☐ 2	☐ 3	☐ 4
c	Advanced practice physiotherapy staff	☐ 1	☐ 2	☐ 3	☐ 4
d	Mental health practitioners	☐ 1	☐ 2	☐ 3	☐ 4
e	Community Link Workers	☐ 1	☐ 2	☐ 3	☐ 4
f	Mental health practitioners	☐ 1	☐ 2	☐ 3	☐ 4
g	Community Treatment and Care staff (e.g. treatment room nurses or phlebotomists)	☐ 1	☐ 2	☐ 3	☐ 4
h	Staff to support (non-COVID-19) vaccination programmes	☐ 1	☐ 2	☐ 3	☐ 4

2. What have been the main advantages of working with additional MDT staff?

____

____

____

____

3. So far, do you feel there have been disadvantages to working with additional MDT staff?

Please ✓ the relevant box on this scale to tell us whether you agree or disagree with each of the following statements.

	1 = Strongly agree	2 = Agree	3 = Slightly agree	4 = Neither agree nor disagree	5 = Slightly disagree	6 = Disagree	7 = Strongly disagree

a	There is not enough clinical space to accommodate the additional staff	☐ 1	☐ 2	☐ 3	☐ 4	☐ 5	☐ 6	☐ 7
b	I now need to spend more time on training and supervising staff	☐ 1	☐ 2	☐ 3	☐ 4	☐ 5	☐ 6	☐ 7
c	The additional staff have taken over some tasks I used to enjoy doing	☐ 1	☐ 2	☐ 3	☐ 4	☐ 5	☐ 6	☐ 7
d	I don't have enough control over what the MDT do as I am not their line manager	☐ 1	☐ 2	☐ 3	☐ 4	☐ 5	☐ 6	☐ 7

4. **What % of your previous clinical work is currently delegated to new MDT staff:** ---------------------------- %

5. **What % of your current clinical work could, in your opinion, be safely delegated to MDT staff:** ---------------------------- %

6. **If there was additional investment, which of these do you regard as <u>the most</u> important?**
(please ✓ only one)

- more GPs ☐ 1
- more MDT staff ☐ 2
- more administrative staff ☐ 3
- Don't know ☐ 4

B) Managing patients with complex needs

9. **Does your Practice <u>schedule longer GP appointments</u> for patients with complex needs (such as those with multimorbidity and/or mental health problems)?**
(*N.B. this does <u>not include</u> routine appointments that overrun*)

- Yes ☐ 1
- No ☐ 2
- Don't know ☐ 3

**10.**	**In your opinion is the new contract improving the care of patients with multimorbidity**	**Yes**	**No**	**Don't know**
a	Who are elderly?	☐ 1	☐ 2	☐ 3

b	Who live in deprived areas?	☐ 1	☐ 2	☐ 3

C) Cluster Working

1. Please ✓ the relevant box on the scale below to rate the extent to which you agree or disagree with these statements about your knowledge and engagement with your GP Cluster.

		Strongly disagree	Disagree	Neutral	Agree	Strongly agree
a	I feel informed about what my GP Cluster is trying to achieve	☐ 1	☐ 2	☐ 3	☐ 4	☐ 5
b	Decisions made by my GP Cluster reflect my views	☐ 1	☐ 2	☐ 3	☐ 4	☐ 5
c	When I make contact, my Practice Quality Lead is responsive to my queries and concerns	☐ 1	☐ 2	☐ 3	☐ 4	☐ 5
d	My GP Cluster is 'owned' by its members and feels like 'our organisation'	☐ 1	☐ 2	☐ 3	☐ 4	☐ 5
e	I can influence the work of my GP Cluster if I choose to	☐ 1	☐ 2	☐ 3	☐ 4	☐ 5

2. Please ✓ the relevant box on the scale below to indicate your opinions on how GP Clusters have affected …

	Decreased a lot	Decreased a little	Not changed	Increased a little	Increased a lot
Your understanding of quality planning (how to set quality improvement goals)	☐ 1	☐ 2	☐ 3	☐ 4	☐ 5
Your understanding of quality improvement (methods and approaches)	☐ 1	☐ 2	☐ 3	☐ 4	☐ 5
Your understanding of quality control (measuring improvement, ensuring safety)	☐ 1	☐ 2	☐ 3	☐ 4	☐ 5
Your understanding of the characteristics of the local population of patients (such as age, deprivation, multimorbidity levels)	☐ 1	☐ 2	☐ 3	☐ 4	☐ 5
The quality of care that you provide	☐ 1	☐ 2	☐ 3	☐ 4	☐ 5
The extent to which you involve patients in decisions about their care, based on what is important to them	☐ 1	☐ 2	☐ 3	☐ 4	☐ 5

**3.** **Are you a <u>Practice Quality Lead</u>?**	*please ✓ the relevant box*<br>Yes ☐ 1 No ☐ 2
… **<u>If Yes</u>**, <u>in total</u> how many hours <u>per month</u> do you spend in this role?	---------------------------- hours

**4.** **Are you a <u>Cluster Quality Lead</u>?**	*please ✓ the relevant box*<br>Yes ☐ 1 No ☐ 2
… **<u>If Yes</u>**, <u>in total</u> how many hours <u>per month</u> do you spend in this role?	---------------------------- hours

… The next 5 questions are for Practice/Cluster Quality Leads <u>Only</u>

**1.** **How often does your Cluster meet?**	*please **delete <u>month</u>** or **<u>year</u>** as applicable*<br>--------------- times per month or year
**2.** **Do other staff (such as Practice Managers and/or Practice Nurses) also attend the Cluster Meetings?**	*please ✓ the relevant box*<br>Yes ☐ 1 No ☐ 2 Don't know ☐ 3

3. **Please ✓ the box on the scale below to rate the extent to which you think your Cluster meetings are...**

	Always	Nearly always	Only sometimes	Hardly ever	Never	Don't know
a Well organised	☐ 1	☐ 2	☐ 3	☐ 4	☐ 5	☐ 6
b Friendly	☐ 1	☐ 2	☐ 3	☐ 4	☐ 5	☐ 6
c Productive	☐ 1	☐ 2	☐ 3	☐ 4	☐ 5	☐ 6
d Well facilitated	☐ 1	☐ 2	☐ 3	☐ 4	☐ 5	☐ 6

4. **Please ✓ the box on the scale below to indicate your opinions on the focus of your Cluster activities at the <u>present time</u>...**

	Always	Nearly Always	Only Sometimes	Hardly Ever	Never	Don't Know
a Its <u>intrinsic role</u> (quality improvement)	☐ 1	☐ 2	☐ 3	☐ 4	☐ 5	☐ 6
b Its <u>extrinsic role</u> (participation in local planning of integrated care)	☐ 1	☐ 2	☐ 3	☐ 4	☐ 5	☐ 6

5. **Please ✓ the relevant box on the scale below to rate the extent to which you feel supported in relation to**

		Fully supported		Almost fully supported		Somewhat supported		Not at all supported		Not relevant	
a	Data	☐	1	☐	2	☐	3	☐	4	☐	5
b	Health intelligence	☐	1	☐	2	☐	3	☐	4	☐	5
c	Analysis	☐	1	☐	2	☐	3	☐	4	☐	5
d	Quality Improvement methods	☐	1	☐	2	☐	3	☐	4	☐	5
e	Advice	☐	1	☐	2	☐	3	☐	4	☐	5
f	Leadership	☐	1	☐	2	☐	3	☐	4	☐	5
g	Evaluation and research	☐	1	☐	2	☐	3	☐	4	☐	5

…… the rest of the questionnaire is for EVERYONE

D. About Your Practice

Please answer each question in this section in relation to ***YOUR CURRENT, MAIN PRACTICE OF EMPLOYMENT***

1. What is your Practice Code or Name --

Please note that this will only be used to obtain routinely available data on your Practice such as patient list size or deprivation ranking.

please ✓ one option for each question)

2. Is your Practice...?

- a multiple GP Partnership ☐ 1
- a single GP Partnership ☐ 2
- Health Board run ☐ 3
- Other *(please specify)* ☐ 4
- Don't know ☐ 5

3. Is your Practice trying to recruit more GPs? Yes ☐ 1 No ☐ 2 Don't know ☐ 3

… If Yes, for how long has the Practice been recruiting?

- < 3 months ☐ 1
- 3-6 months ☐ 2
- 7-12 months ☐ 3
- > 12 months ☐ 4
- Don't know ☐ 5

4. Is your Practice trying to recruit more MDT staff? Yes ☐ 1 No ☐ 2 Don't know ☐ 3

… If Yes, for how long has the Practice been recruiting?

- < 3 months ☐ 1
- 3-6 months ☐ 2
- 7-12 months ☐ 3
- > 12 months ☐ 4
- Don't know ☐ 5

(please ✓ one option for each sub-question)

5. **In the past 18 months, has your Practice been involved in education or training of …**

Undergraduate students	Yes	☐ 1	No	☐ 2	Don't know	☐ 3
Foundation doctors	Yes	☐ 1	No	☐ 2	Don't know	☐ 3
GP Registrars/ST3	Yes	☐ 1	No	☐ 2	Don't know	☐ 3
Other health professionals (such as ANP, MSK physios)	Yes	☐ 1	No	☐ 2	Don't know	☐ 3
Other (please specify)	Yes	☐ 4	________________			

E. About You

1. **Please ✓the relevant box or write in the space provided**

a Do you identify as being ….

Male ☐ 1 Female ☐ 2 Other ☐ 3

b What age are you? ---------------- years

c How would you classify your ethnic background?

		White	☐ 1	Pakistani	☐ 2
Black-African	☐ 3	Black-Other	☐ 4	Indian	☐ 5
Chinese	☐ 6	Mixed	☐ 7	Other (specify below)	☐ 8

----

2. **Please ✓ the relevant box that indicates the <u>likelihood</u> that you will make the following changes.**

		None	Slight	Moderate	Considerable	High
a	Increase your work hours within <u>5 years</u>	☐ 1	☐ 2	☐ 3	☐ 4	☐ 5
b	Reduce your work hours within <u>5 years</u>	☐ 1	☐ 2	☐ 3	☐ 4	☐ 5
c	Continue medical work but outside the UK within <u>5 years</u>	☐ 1	☐ 2	☐ 3	☐ 4	☐ 5
d	Leave direct patient care within <u>5 years</u>	☐ 1	☐ 2	☐ 3	☐ 4	☐ 5
e	Leave medical work entirely within <u>5 years</u>	☐ 1	☐ 2	☐ 3	☐ 4	☐ 5

3. **If you are planning to leave direct patient care or medical work entirely within 5 years, have you taken any preparatory steps towards this?**

Yes ☐ 1

No ☐ 2

	(such as investigated pension, consulted with partners, looked for another job)	Not applicable ☐ 3
**4.**	**At what age do you plan to retire from medical practice?**	____ years
	... and what is the likelihood of this happening?	Definit ☐ Very ☐ Quite ☐ Unlikel ☐
**5.**	**Would you recommend general practice as a specialty for new medical graduates?**	Yes ☐ No ☐

6. **Please ✓ the relevant box on the scale below to rate overall how satisfied you are with life**

1 = Extremely satisfied **7 = Extremely dissatisfied**

☐ 1 ☐ 2 ☐ 3 ☐ 4 ☐ 5 ☐ 6 ☐ 7

MANY THANKS FOR YOUR HELP WITH THIS SURVEY.

Table S1. Representativeness of GP survey and comparison with 2018 GP survey

	Scotland (2022)	GP survey (2023)	GP survey (2018)
**Age (years)**			
< 35	337 (7%)	108 (8%)	229 (10%)
35-44	1864 (41%)	403 (31%)	775 (34%)
45-54	1454 (32%)	510 (39%)	806 (36%)
55-64	789 (18%)	260 (20%)	376 (19%)
65 +	66 (2%)	13 (1%)	19 (1%)
**Gender**	3217 (61%)	788 (57%)	1433 (58%)
**Ethnicity (white)**	-	1254 (92%)	2232 (93%)
**GP Partner**	3236 (72%)	1143 (83%)	2048 (84%)
**Deprivation**	903 (20%)	276 (20%)	492 (20%)
**Urban locality**	3296 (73%)	957 (69%)	1689 (69%)

Deprivation is calculated as the proportion of patients in the most deprived quintile of all practices in Scotland, calculated from the percentage of registered patients who are living in the top 15% most deprived areas based on the Scottish Index of Multiple Deprivation (SIMD). Urban locality is based on the proportion of registered patients living in urban conurbations as defined by the Scottish Government. Publicly available data on all GPs in Scotland (2022) was accessed from Public Health Scotland; https://publichealthscotland.scot/publications/general-practice-gp-workforce-and-practice-list-sizes/general-practice-gp-workforce-and-practice-list-sizes-2012-2022/

Figure S1. Distribution of females and white ethnicity by age in 2018 and 2023 surveys

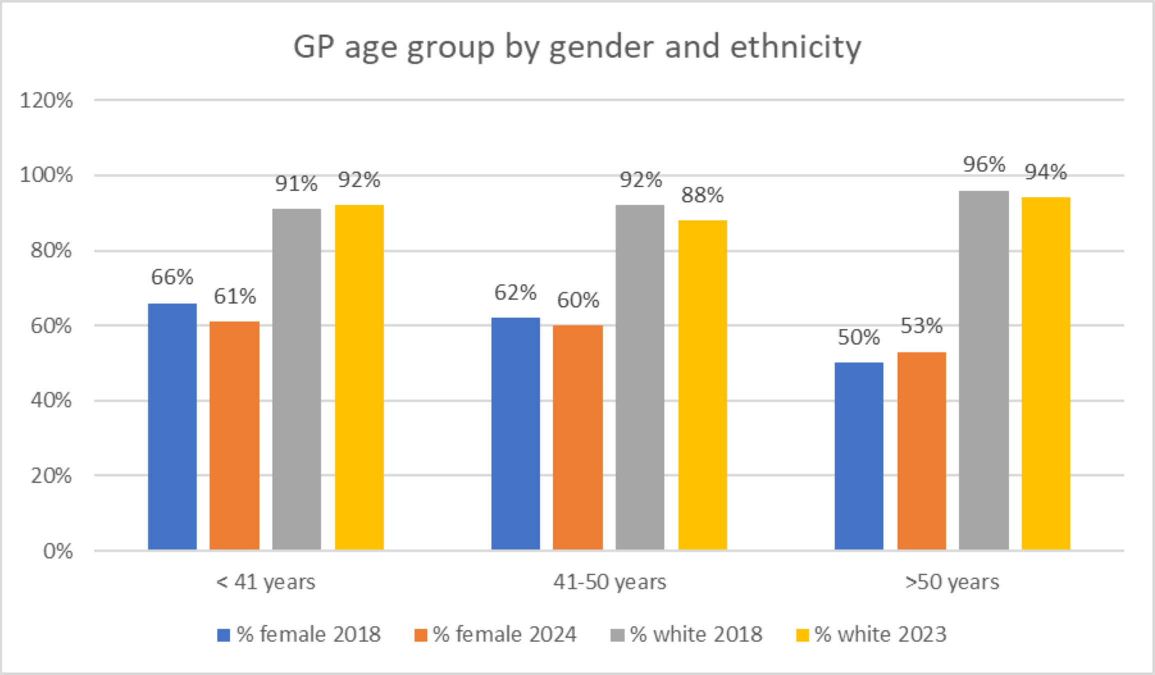

Figure S2. Distribution of ethnicity, urbanicity and deprivation by gender in 2018 and 2023 surveys

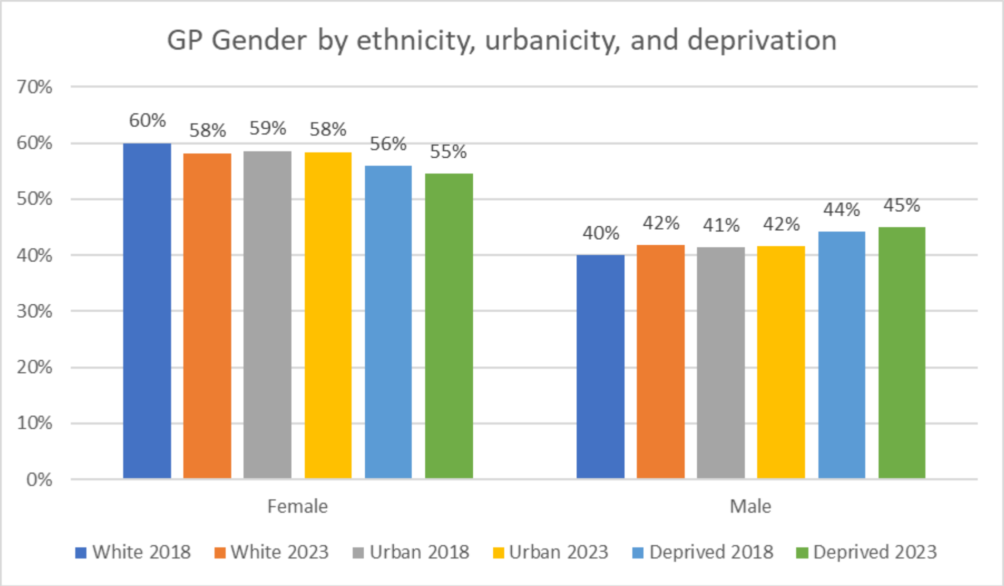

Table S2. Individual domains of GP job satisfaction in 2023 and 2018

	**GP survey 2023**	**GP survey 2018**	P value
**Satisfaction with:** Mean (SD)			
Physical working conditions	5.42 (1.42)	5.49 (1.46)	0.018
Freedom to choose method of working	5.48 (1.21)	5.33 (1.29)	0.002
Colleagues and fellow workers	6.18 (1.18)	6.09 (1.15)	0.016
Recognition for good work	4.66 (1.56)	4.72 (1.55)	0.167
Amount of responsibility	5.45 (1.47)	5.48 (1.45)	0.460
Remuneration	4.77 (1.63)	4.92 (1.58)	0.004
Opportunities to use abilities	5.47 (1.27)	5.36 (1.27)	0.004
Hours of work	4.75 (1.71)	4.51 (1.68)	<0.001
Variety in job	5.74 (1.17)	5.59 (1.23)	0.001
Overall feelings about job	5.07 (1.24)	5.01 (1.35)	0.630

Satisfaction items measured on a 7-point scale (1= extremely dissatisfied, 7= extremely satisfied). Non-parametric (Mann-Witney) test was used to calculate p values.

Table S3. Individual domains of GP job pressure in 2023 and 2018

	**GP Survey 2023**	**GP survey 2018**	P value
**Pressure from:** Mean (SD)			
Increased patient demand	4.10 (0.89)	4.0 (0.92)	0.001
Problem patients	3.82 (0.95)	3.78 0.97)	0.433
Early discharge from hospital	3.71 (1.04)	3.58 (0.99)	<0.001
Patient complaints	3.28 (1.17)	3.19 (1.14)	0.023
Insufficient time	3.93 (1.04)	3.91 (1.14)	0.418
Interruptions during consultations	2.80 (1.14)	2.62 (1.10)	<0.001
Unrealistic expectations	3.49 (1.22)	3.30 (1.22)	<0.001
Insufficient resources	3.43 (1.21)	3.19 (1.17)	<0.001
Long hours	3.49 (1.21)	3.48 (1.16)	0.836
Paper work	3.77 (1.04)	3.78 (0.99)	0.852
External bodies	3.01 (1.10)	3.03 (1.05)	0.366
Quality linked payments	2.47 (1.11)	2.59 (1.06)	<0.001
Finding locums	3.44 (1.42)	3.61 (1.43)	<0.001
Negative media	3.86 (1.17)	3.33 (1.25)	<0.001
Increasing workload	4.24 (0.93)	4.16 (0.94)	<0.001

Work pressure is measured on a 5-point scale (1=no pressure, 2=slight pressure, 3=moderate pressure, 4=considerable, 5= high pressure). Non-parametric (Mann-Witney) test was used to calculate p values.

Table S4. Individual domains of GP positive job attributes in 2023 and 2018

	**GP survey2023**	**GP survey 2018**	**P value**
**Positive job attributes: M**ean (SD)			
Choice in how I do my job	3.63 (0.87)	3.47 (1.00)	<0.001
Choice in what I do at work	3.20 (0.97)	3.14 (1.06)	0.014
Working time is flexible	2.88 (0.97)	2.87 (1.19)	0.340
Interesting variety in things	3.99 (0.75)	4.03 (0.81)	0.600
Involved in deciding on changes	3.36 (1.14)	3.19 (1.17)	<0.001
I always know my work responsibilities	3.92 (0.81)	3.79 (0.91)	<0.001
Consulted about changes	3.19 (1.17)	3.09 (1.19)	<0.001
Clear feedback about performance	2.78 (0.98)	2.74 (1.03)	0.027
I decide how to do my work	3.21 (0.10)	3.19 (1.07)	0.074
Changes have led to better patient care (D)	2.52 (1.04)	2.62 (1.05)	-0.098
Patients trust my generalist skills	4.01 (0.64)	4.07 (0.74)	-0.084

Job attributes are measured on a 5-point scale (1=strongly disagree, 2=disagree, 3=neutral, 4=agree, 5=strongly agree). Non-parametric (Mann-Witney) test was used to calculate p values.

Table S5. Individual domains of GP negative job attributes in 2023 and 2018

	**GP survey 2023**	**GP survey 2018**	**P value**
**negative job attributes:** Mean (SD)			
I have to work very fast	4.14 (0.82)	4.11 (0.89)	0.092
I have to work very intensively	4.34 (0.755)	4.37 (0.792)	0.787
Patient complexity has increased	4.71 (0.52)	4.68 (0.42)	0.028
I do not have time to do all my work	3.79 (1.09)	3.81 (1.09)	0.750
Relationships at work are strained	2.25 (1.05)	2.28 (1.10)	0.502
I am required to do unimportant tasks	3.88 (1.00)	3.93 (1.02)	0.330

Job attributes are measured on a 5-point scale (1=strongly disagree, 2=disagree, 3=neutral, 4=agree, 5=strongly agree). Non-parametric (Mann-Witney) test was used to calculate p values.

Table S6. GPs views on Clusters in 2023 and 2018

Mean (SD)	GP survey 2023	GP survey 2018	P value
**Quality Leads views**			
Cluster meetings are:			
Well organised	1.94 (0.82)	1.96 (0.84)	0.735
Friendly	1.43 (0.62)	1.47 (0.59)	0.233
Productive	2.53 (1.00)	2.50 (0.96)	0.648
Well-facilitated	1.97 (0.90)	2.01 (0.87)	0.376
Total average score for meetings	1.95 (0.68)	1.98 (0.61)	0.578
Cluster activities:			
Focus on intrinsic role	2.36 (0.88)	2.24 (0.80)	0.083
Focus on extrinsic role	2.97 (1.58)	2.75 (1.01)	0.062
Extent of support in relation to:			
Data	3.02 (0.80)	2.94 (0.753)	0.051
Health intelligence	3.15 (0.82)	3.09 (0.73)	0.068
Analysis	3.22 (0.81)	3.12 (0.72)	0.006
QI methods	2.92 (0.80)	3.05 (0.74)	0.041
Advice	2.92 (0.82)	3.00 (0.74)	0.191
Leadership	2.85 (0.85)	3.00 (0.81)	0.009
Evaluation and research	3.29 (0.72)	3.29 (0.69)	0.831
Total average score for support	3.07 (0.66)	3.06 (0.63)	0.578
**All other GPs views**			
Cluster knowledge and engagement:			
Informed about Cluster	3.27 (1.11)	3.25 (1.11)	0.488
Decisions reflect my views	3.19 (0.93)	3.10 (0.95)	0.003
PQL responsive to queries	3.74 (0.87)	3.64 (0.86)	<0.001
Cluster owned by its members	3.09 (1.00)	3.06 (1.00)	0.328
Can influence work of Cluster	3.23 (1.03)	3.23 (1.00)	0.876
Total average score CKE	3.30 (0.82)	3.25 (0.89)	0.082
Understanding of QI:			
Quality planning	3.30 (0.67)	3.14 (0.69)	<0.001
Quality improvement	3.32 (0.67)	3.15 (0.68)	<0.001
Quality control	3.29 (0.64)	3.12 (0.65)	<0.001
Local population	3.32 (0.66)	3.21 (0.65)	<0.001
Quality of care	3.27 (0.60)	3.13 (0.55)	<0.001
Shared-decision making with patients	3.16 (0.43)	3.23 (1.00)	<0.001
Total average score CQI	3.27 (0.82)	3.14 (0.51)	<0.001

Cluster meetings and activities items are measured on a 5-point scale; (1= always, 2= nearly always, 3=sometimes, 4=hardly ever, 5 = never).
Extent of support measured on a 4-point scale; 1=fully supported; 2=almost fully supported; 3=somewhat supported; 4=not at all supported.
Other GPs knowledge about clusters scored on a 5 point scale; 1=strongly disagree, 2=disagree, 3=neutral, 4=agree, 5= strongly agree.
Understanding of QI scored on a 5 point scale; 1=decreased a lot, 2=decreased a little, 3=not changed, 4= increased a little, 5= increased a lot.
Non-parametric (Mann-Witney) test was used to calculate p values.

Table S7. Disadvantages of new MDT staff

	Strongly agree	Agree	Slightly agree	Neutral	Slightly disagree	Disagree	Strongly disagree
Lack of clinical space	316 (22.9%)	303 (22.0%)	319 (23.2%)	133 (9.7%)	51 (3.7%)	172 (12.5%)	83 (6.0%)
Training and supervision	200 (14.6%)	330 (24.1%)	308 (22.4%)	200 (14.6%)	78 (5.7%)	184 (13.4%)	72 (5.2%)
Taken over tasks I enjoyed	78 (5.7%)	195 (14.2%)	181 (13.2%)	249 (18.1%)	165 (12.0%)	341 (24.8%)	165 (12.0%)
Lack of control over their job	483 (35.2%)	301 (21.9%)	194 (14.1%)	187 (13.6%)	62 (4.5%)	88 (6.4%)	59 (4.3%)

Table S8. GPs views in changes in the last 12 months in 2023 and 2018

Mean (SD)	**GP survey 2023**	**GP survey 2018**	**P value**
NHS services for your patients	2.04 (1.09)	2.37 (1.02)	**<0.001**
NHS services in your local area	1.60 (0.84)	2.00 (0.89)	**<0.001**
Practice workload	1.78 (0.82)	2.03 (0.87)	**<0.001**
Personal workload	1.93 (0.91_	2.11 (0.93)	**<0.001**
Practice sustainability	2.26 (1.03)	2.45 (1.03)	**<0.001**
Job satisfaction	2.43 (1.00)	2.58 (0.93)	**<0.001**

Measured on a 5-point scale; 1= worsened a lot, 2= worsened a little, 3= not changed, 4=improved a little, 5= improved a lot. Non-parametric (Mann-Witney) test was used to calculate p values.

Figure S3. GP practice involvement in education and training in 2023 and 2018

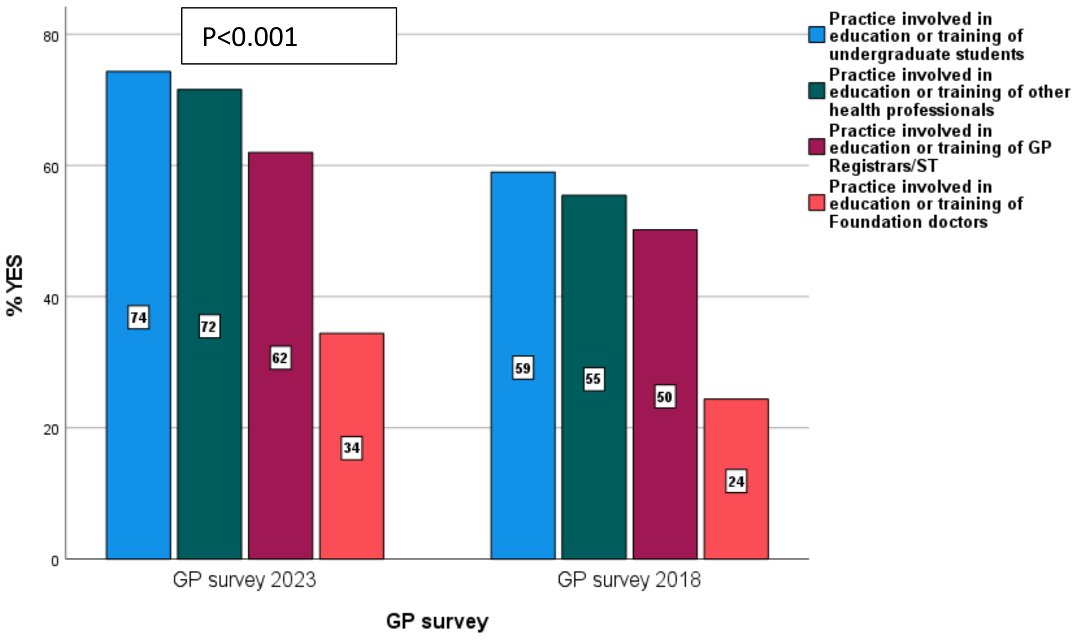

Non-parametric (Mann-Witney) test was used to calculate p values.

Comparison of GPs who did or did not complete both surveys

We carried out an additional analysis of GPs who responded to the surveys at both time points. There were 811 such GPs (33% of the 2018 respondents and 59% of the 2023 respondents). The 811 GPs who responded to both surveys were significantly younger, and worked in less deprived practices than the GPs in the 2018 survey who did not respond to the 2023 survey, as shown below, in the Table S9. Thus, this group of 811 GPs were less nationally representative than the 2018 and 2023 respondents as a whole.

Table S9. GP and practice demographics of GPs in the 2018 survey who did or did not also take part in the 2023 survey

	**GPs in 2018 survey who did not take part in 2023 survey**	**GPs in the 2018 survey who did take part in the 2023 survey**	**P value**
**Age in years:** Mean (SD)	47.2 (9.10)	44.6 (7.31)	P<0.001
**Age distribution (years):** n (%)		**N (%)**	
< 35	149 (9.7%)	80 (10.4%)	P<0.001
35-44	475 (30.9%)	300 (39.1%)	
45-54	501 (32.6%)	315 (41.0%)	
55-64	395 (25.7%)	71 (9.2%)	
65 +	17 (1.1%)	2 (0.3%)	
**Gender (female):** n (%)	937 (57.0%)	496 (60.9%)	0.065
**Ethnicity (white):** n (%)	1501 (93.3%)	731 (91.8%)	0.194
**GP Partner;** (n (%)	1375 (84.1%)	673 (83.0%)	0.482
**Works ≥ 7 session per week**: n (%)	894 (54.5%)	451 (55.5%)	0.641
**Remote and Rural locality:** n (%)	243 (14.4%)	113 (14.0%)	0.805
**Deprivation**: n (%)			
Quintile 1: Least Deprived	306 (18.8%)	173 (21.5%)	P=0.031
Quintile 2	323 (19.9%)	173 (21.5%)	
Quintile 3	328 (20.2%)	153 (19.0%)	
Quintile 4	321 (19.8%)	160 (19.9%)	
Quintile 5: Most Deprived	346 (21.3%)	146 (18.1%)	

We then compared the GP working life domains, and the future job intentions in 2023 and 2018, and have found some minor differences between the full sample and the paired sample, as shown in Table S10. As can be seen, in the current working life domains, job satisfaction in 2023 and 2018 remained statistically insignificant in both groups, and positive job attributes increased in both. However, job pressures did not show a significant increase in the paired sample whereas it did in the full sample surveys, and negative job pressure improved (decreased) significantly in 2023 versus 2018 in the paired sample but not in the full sample comparison. In the future job intensions items, the paired sample showed highly significant differences (p<0.001) between two survey years for all items, that is in 2023 compared with 2018 GPs were less likely to planning to increase their hours, more likely to be planning to decrease their hours, leave the UK, leave direct patient care and leave medical work entirely. In the full sample comparison, only decreasing hours and leave direct patient care were significantly different. It should be noted though that the difference in the mean scores were very small in all groups for working life domains and future intentions, with the intention to decrease hours showing the largest mean difference. In terms of effect sizes (Cohen's d) for the working life domains (which were normally distributed) these were all <0.2 in both the paired

group and the full sample group when comparing the two survey years, suggesting that the differences in mean scores, whether statistically significant or not, were minimal and likely to be of negligible importance.

Table S3. GPs current working life and future work intentions in 2023 and 2018 (all respondents) and respondents completing both surveys (paired)

	**GP Survey 2023 Full sample (n=1378)**	**GP survey 2018 Full sample (n=2454)**	**Mean difference**	**P value (unpaired tests)**	**GP survey 2023 paired sample (n=811)**	**GP survey 2018 paired sample (n=8111)**	**Mean difference**	**P value (paired tests)**
**Current working life:**								
Job satisfaction	5.31 (0.95)	5.26 (0.99)	0.05	0.128	5.29 (0.97)	5.28 (0.97)	0.01	0.876
Job pressures	3.52 (0.78)	3.45 (0.74)	0.07	**0.004**	3.57 (0.76)	3.54 (0.75)	0.03	0.355
Positive job attributes	3.34 (0.54)	3.29 (0.59)	0.05	**0.011**	3.34 (0.54)	3.20 (0.56)	0.16	**<0.001**
Negative job attributes	3.85 (0.56)	3.86 (0.58)	0.01	0.594	3.91 (0.55)	4.05 (0.64)	-0.14	**<0.001**
**Future intentions:**								
Increase hours	1.41 (0.83)	1.49 (0.95)	-0.08	0.090	1.32 (0.75)	1.53 (0.95)	-0.21	**<0.001**
Decrease hours	3.08 (1.56)	2.81 (1.56)	0.27	**<0.001**	3.29 (1.56)	2.60 (1.46)	0.69	**<0.001**
Work outside UK	1.44 (0.92)	1.42 (0.85)	0.02	0.923	1.39 (0.87)	1.46 (0.89)	-0.07	**<0.001**
Leave direct patient care	2.39 (1.53)	2.24 (1.52)	0.15	**0.008**	2.58 (1.59)	1.86 (1.26)	0.72	**<0.001**
Leave medical work entirely	2.24 (1.52)	2.18 (1.53)	0.06	0.060	2.46 (1.61)	1.73 (1.20)	0.73	**<0.001**
